# Phylogeny, distribution and potential metabolism of candidate bacterial phylum KSB1

**DOI:** 10.7717/peerj.13241

**Published:** 2022-04-12

**Authors:** Qingmei Li, Yingli Zhou, Rui Lu, Pengfei Zheng, Yong Wang

**Affiliations:** 1 Institute of Deep Sea Science and Engineering, Chinese Academy of Sciences, Sanya, China; 2 University of Chinese Academy of Sciences, Beijing, China; 3 Institute for Marine Engineering, Shenzhen International Graduate School, Tsinghua University, Shenzhen, China

**Keywords:** Marine sediment, Bioreactor, KSB1, Phylogenomics, Short-chain hydrocarbon

## Abstract

Candidate phylum KSB1 is composed of uncultured bacteria and has been reported across various environments. However, the phylogeny and metabolic potential of KSB1 have not been studied comprehensively. In this study, phylogenomic analysis of KSB1 genomes from public databases and eleven metagenome-assembled genomes (MAGs) from marine and hydrothermal sediments revealed that those genomes were clustered into four clades. Isolation source and relative abundance of KSB1 genomes showed that clade I was particularly abundant in bioreactor sludge. Genes related to dissimilatory reduction of nitrate to ammonia (DNRA), the last step of denitrification converting nitrous oxide to nitrogen and assimilatory sulfur reduction were observed in the expanded genomes of clade I, which may due to horizontal gene transfer that frequently occurred in bioreactor. Annotation and metabolic reconstruction of clades II and IV showed flagellum assembly and chemotaxis genes in the genomes, which may indicate that exploration and sensing for nutrients and chemical gradients are critical for the two clades in deep-sea and hydrothermal sediment. Metabolic potentials of fatty acids and short-chain hydrocarbons utilization were predicted in clades I and IV of KSB1. Collectively, phylogenomic and metabolic analyses of KSB1 clades provide insight into their anaerobic heterotrophic lifestyle and differentiation in potential ecological roles.

## Introduction

Uncultured candidate division KSB1 is a bacteria phylum that had not been studied well yet. Monophyleticity of KSB1 was firstly detected in marine coastal with sulfur-rich black mud as shown by 16S rRNA amplicon sequencing and phylogenetic analysis (Tanner et al., 2000). In an anoxic treatment lagoon, the relative abundance of the phylum KSB1 was up to 4% (Cardinali-Rezende et al., 2012), which suggested that KSB1 might be enriched in an anoxic environment. In microbial mat of a solar saltworks, KSB1 was the most abundant in an anoxic zone with a low-H_2_S rather than high-H_2_S concentration (Ley et al., 2006), suggesting impact of H_2_S concentration on KSB1. Overall, KSB1 were widely distributed across different habitats such as marine coastal, hypersaline microbial mat, cave sediment, aquifer and swine sludge (Anantharaman et al., 2016; Cardinali-Rezende et al., 2012; Ley et al., 2006; Tanner et al., 2000; Yasir, 2018), indicating their high adaptive flexibility and biodiversity.

KSB1 bacteria are potentially able to encode genes involved in versatile metabolisms. The first genomics study of KSB1 from estuary sediment indicated the capacity for carbohydrate hydrolysis and beta-oxidation (Baker et al., 2015). The KSB1 genomes from hydrothermal sediments contain the genes involved in anaerobic degradation of hydrocarbon and activating polycyclic aromatic hydrocarbons (PAHs) and alkanes with fumarate addition mechanism (Dombrowski et al., 2017). Moreover, the KSB1 phylum inhabiting wetland sediment might conduct isopropanol-butanol-ethanol fermentation (Dalcin Martins et al., 2019). These heterotrophic metabolisms utilizing a variety of organic compounds reflect high adaptive flexibility of KSB1 in diverse ecological niches, which may indicate the importance of KSB1 in recycling of organic debris and hydrocarbons in anoxic environments. All the capacities of KSB1 seem to be a result of high genomic diversity and an indicator of their potentially important roles in ecosystems. However, a comprehensive phylogenomic and metabolic analysis of KSB1 members has not been conducted yet.

In this study, a total of 44 nonredundant high quality KSB1 genomes, including 11 MAGs obtained by this study, were analyzed. A phylogenomic tree revealed that the KSB1 genomes were distributed roughly across four clades. MAGs coverage and 16S rRNA genes of KSB1 were used for exploring their distribution in different niches, particularly in global oceans. Metabolic reconstruction revealed differentiation of gene content and metabolism for anaerobic heterotrophic mode of life among the four clades of KSB1 derived from diverse niches and their potential roles in biogeochemical cycles. We also revealed remarkable genomic expansion of KSB1 clade I with additional genes under the impact of the complex substrates and microbial community in bioreactor.

## Materials and Methods

### Collection of KSB1 genomes

A total of 42 nonredundant KSB1 genomes were downloaded from GTDB, NCBI, JGI databases and published papers (Table S1) (November, 2020) and were filtered with following cutoff values: completeness score ≥ 50%, contamination rate ≤ 10% and QS > 50 (QS = completeness-5*contamination) (Almeida et al., 2019). KSB1 genomes were also binned from the metagenomes of the Mariana sediments collected 5,400 to 10,911 m depths during *R/V* DY37-II, TS01 and TS03 (Cui et al. 2021). Fastp (Chen et al., 2018a) (v.0.20.0) was used for the quality control of metagenome raw data. Repeated Illumina sequences were removed by Fastuniq (Xu et al., 2012) and the clean reads were assembled with SPAdes (v.3.13) (Bankevich et al., 2012). Contigs >2,000 bp were used for genome binning with MetaWRAP (v.1.2) integrated with three binning tools, followed by a treatment with bin_refinement module (Uritskiy, DiRuggiero & Taylor, 2018). The MAGs with qualified completeness (higher than 50%) and contamination (lower than 10%) were selected by CheckM (Parks et al., 2015). The MAGs affiliated with KSB1 were selected from the classification result of GTDB-tk (v.1.4.0) software (Chaumeil et al., 2019) integrated with GTDB release95 database. One KSB1 genome named vent-69 had been obtained from a Mid-Atlantic hydrothermal sediment metagenome (accession: SAMN10350645).

### Calculation of KSB1 relative abundance in different niches

16S rRNA gene sequences of the KSB1 genomes were extracted to create a dataset. The 16S miTags of the Tara Ocean data were downloaded from http://ocean-microbiome.embl.de/data/16SrRNA.miTAGs.tgz. KSB1 16S miTags (metagenomic Illumina tags) were identified from the Tara Ocean miTags (Moran, 2015) by BLASTn (Gish & States, 1993) (v.2.9.0) (-evalue 1e–05) against the 16S rRNA dataset of KSB1. The mapped KSB1 16S miTags with 97% identity and at least 100 bp in length were further selected from the BLASTn result as KSB1 16S miTags for calculation of their relative abundance in the marine water samples. Raw data of public metagenomes (fastq files) were downloaded from NCBI and were subjected to quality control as described above. The relative abundance of KSB1 MAGs in the metagenomes was calculated by coverM (https://github.com/wwood/CoverM, -m relative_abundance; -min-read-aligned-length 50; -min-read-percent-identity 0.99; -min-covered-fraction 0) after mapping with bwa mem (Li & Durbin, 2009) (default parameter) and sorting with samtools (default parameter) (Li et al., 2009).

### Gene annotation and metabolic reconstruction

Genomes from public databases and this study were used for gene annotation. Open reading frames (ORFs) were predicted by Prodigal (Hyatt et al., 2010) (v.2.6.3) and were searched against KEGG (Kyoto Encyclopedia of Genes and Genomes) database (release 92) by Kofamscan (Aramaki et al., 2020) (v.1.0.0; -f mapper), COG (Cluster of Orthologous Groups of proteins) database (COG_2019_v11.0) (Tatusov et al., 2000) by BLASTp (Eddy, 1995) (BLAST+ v.2.9.0) (-evalue 1e–05) and CAZy database (dbCAN-HMMdb-V7) by hmmscan (Eddy, 1995) (v.3.2.1) with default settings. Phage was predicted in http://phaster.ca/.

### Phylogenetic analysis of genomes and proteins

Single copy marker proteins in the KSB1 MAGs and reference genomes were identified with GTDB-tk classify_wf (Chaumeil et al., 2019). The marker proteins were filtered by their presence in 80% of the MAGs. The selected marker proteins were concatenated and used for reconstruction of a phylogenomic tree with iqtree2 (Minh et al., 2020) (v.2.1.0; -m MFP) after multiple sequence alignment using MAFFT (Katoh & Standley, 2013) (v7.453) and alignment optimization using trimAl (Capella-Gutierrez, Silla-Martinez & Gabaldon, 2009) (v.1.4). A phylogenomic tree of the genomes from FCB superphylum (Table S2) was constructed by iqtree2 with MFP model using 43 conserved proteins selected by checkM (Parks et al., 2015).

The amino acid sequences encoded by *narG*, *nrfA* and *nosZ* identified in KSB1 genomes were searched against the NCBI_nr database. The most similar homologous sequences from different phyla were retrieved for phylogenetic tree construction as described above but with MFP+LM model. The protein sequences of NosZ were obtained from FunGene database (Fish et al., 2013) and clustered by CD-HIT (Li & Godzik, 2006) (-c 0.76 -A 0.8). An unroot phylogenetic tree was built for KSB1 as mentioned above.

### MAG availability

The MAGs of KSB1 binned from the Mariana sediments and the Mid-Atlantic Ridge hydrothermal sediment were submitted to the National Omics Data Encyclopedia (NODE) with OEP002159 as project accession number and OER184014 as run ID.

## Results and discussion

### Phylogenomics of KSB1

A total of 89 genomes of KSB1 were collected from public databases, including GTDB, JGI and NCBI. In addition, 14 KSB1 genomes provided by published papers were recruited manually as well (Table S1). Fifteen KSB1 MAGs retrieved from metagenomes of the Mariana sediments with depths ranging from 5,400 to 10,953 m and the Mid-Atlantic hydrothermal vent by this study were added into the KSB1 dataset. After dereplication and quality control for the 118 genomes, 44 of them were retained for further study (Table S1), which included 11 MAGs from this study in size of 2.87~5.37 Mbp (Table 1).

**Table 1:** KSB1 MAGs binned from Mariana Trench and Mid-Atlantic hydrothermal sediment.

MAG id	Depth (m)	Genome size (Mbp)	GC (%)	Com. (%)	Con. (%)	No. contigs	No. ORFs
B3T3L14	10,911	4.62	39	97.80	0.00	216	4,094
B23T1B10	8,638	5.37	39	93.41	4.46	395	4,947
vent-69	1,720	3.69	50	96.64	1.10	328	3,121
B11D1T2	5,533	4.11	44	87.45	4.95	885	4,391
B13T1L6	7,850	4.48	44	94.99	7.14	438	4,347
B16T1L6	7,850	2.87	52	76.69	0.00	396	2,761
B24T1B10	8,638	3.47	44	75.94	2.26	542	3,565
B4MC02	5,400	4.09	44	95.54	6.59	617	4,149
B70T1B8	7,143	3.09	43	95.54	7.41	411	3,137
B77T1B5	7,061	4.75	44	96.64	6.04	503	4,660
B79T1L10	10,911	4.66	45	85.65	4.68	822	4,744

**Notes:**

vent-69 was a KSB1 MAG binned from the Mid-Atlantic Ridge hydrothermal sediment metagenome data downloaded from NCBI with the SRA number SAMN10350645. All the others were binned from metagenomes for the Mariana Trench sediments.

Com., completeness; Con., contamination.

**Figure 1:**
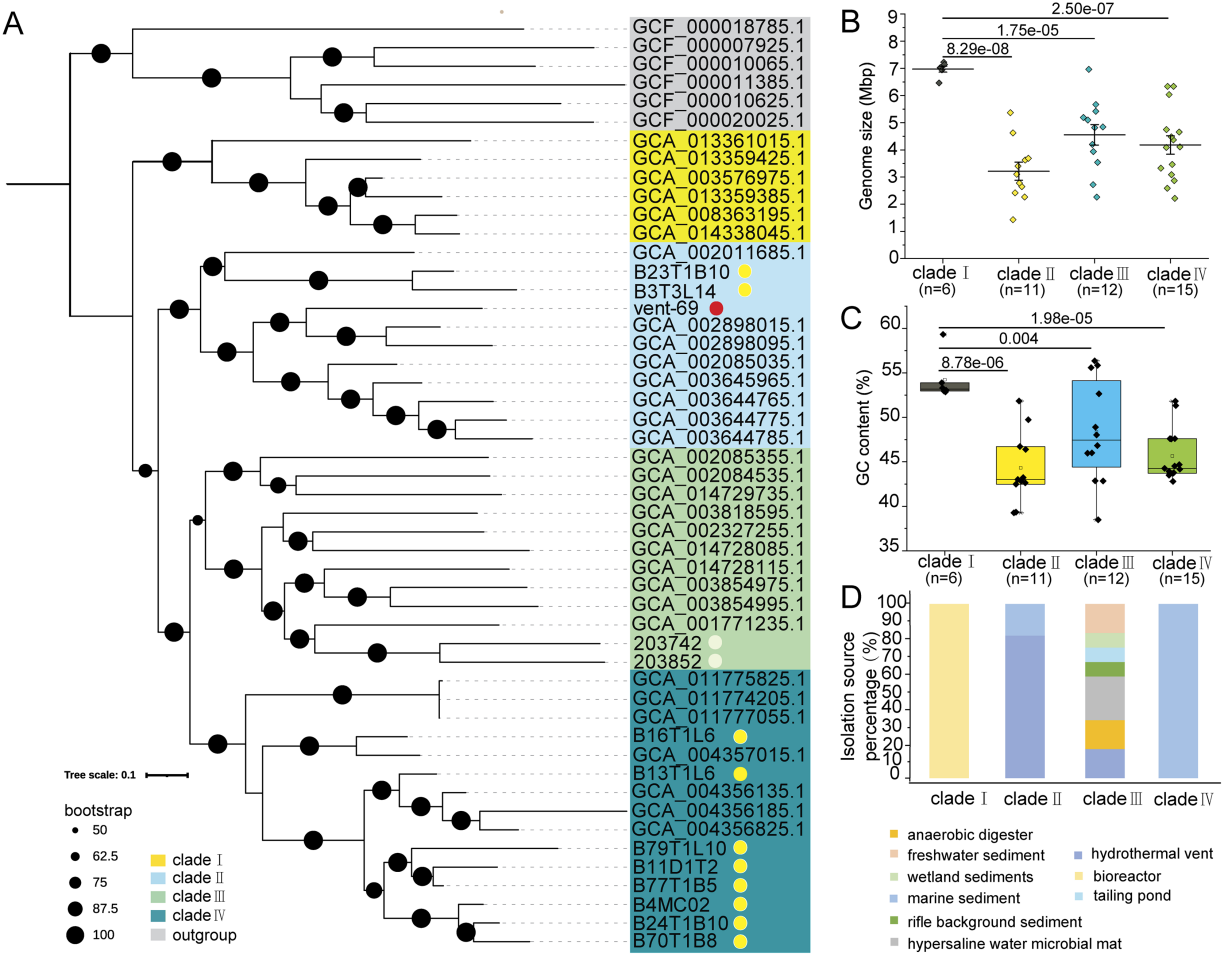
Phylogenomics analysis, genomic traits and distribution of KSB1. Phylogenomics tree was constructed by using deduced conserved proteins of KSB1 MAGs (A). The MAGs binned from the Mariana sediment metagenomes were associated with a yellow dot; KSB1 MAGs downloaded from JGI were marked with a white dot and the MAG from the hydrothermal sediment was associated with a red dot. Genome size (B) and GC content (C) were plotted and compared among the clades (*t*-test; *p* values were shown between groups). Distribution (environment source) of different KSB1 clades (D).

The phylogenomic tree constructed by using 39 conserved marker proteins displayed four phylogenetic clades of KSB1, which were then named as ‘clades I–IV’ (Fig. 1A). The 11 KSB1 MAGs from this study were distributed into two clades (clade II and clade IV). Particularly, most KSB1 MAGs of the Mariana sediments were grouped into clade IV. According to the microbial phylogenetic tree of hydrothermal sediments (Dombrowski, Teske & Baker, 2018) and a previous phylogenetic inference of KSB1 (Youssef et al., 2019), KSB1 might be affiliated with the Fibrobacteres-Chlorobi-Bacteroidetes (FCB) superphylum or be a sister phylum of the superphylum. To examine this hypothesis, we constructed a phylogenomic tree using some high-quality genomes of the FCB superphylum with Proteobacteria and Therrabacteria serving as the outgroup (Table S2). The topological structure of the tree showed that KSB1 was placed into one monophyletic branch within the FCB superphylum and was adjacent to SAR406 (Huang & Wang, 2020) and ‘*Candidatus* Tianyabacteria’ (Cui et al., 2021) (Fig. S1).

The genome size and GC content of the 44 KSB1 MAGs were calculated. The mean genome size of clade I with six MAGs was around 7 Mbp, which was significantly larger than other three clades (t-test; *p* < 0.001) (Fig. 1B). The median GC content of clade I was 52.50%, significantly higher when compared to other clades with a t-test (*p* < 0.001 for clade II with 11 MAGs; *p* = 0.004 for clade III with 12 MAGs; *p* < 0.001 for clade IV with 15 MAGs) (Fig. 1C). The isolation sources of the 44 MAGs were summarized to demonstrate distribution specificity of KSB1 clades. These MAGs of clade I were uniquely from bioreactors, while clade IV was exclusively from marine sediments (Fig. 1D). In contrast, MAGs of clade III were isolated from different environments including freshwater sediment, wetland sediment and tailing pond (Fig. 1D), indicating that clade III is broadly distributed. For the KSB1 MAGs of clade II, 18.18% genomes were obtained from marine sediments and 81.82% genomes were identified in hydrothermal vent environments (Fig. 1D).

A previous study has indicated that genome size might correlate with GC content (Wu et al., 2012). The large genome size of clade I may be ascribed to a large number of genes and mobile genetic elements in the genomes. It has been reported that mobile DNA density increased when the genome size was enlarged (Newton & Bordenstein, 2011). Prediction of transposases against KEGG and COG databases revealed that these genes were more frequently present in clades I and IV (Table S3). Therefore, transposases might be one of the factors driving the genome expansion of clade I (Fig. 1B). Phage or CRISPR/Cas arrays might be an effective mobile element (Al-Shayeb et al., 2020; Ali et al., 2020; Sanderson et al., 2020) for the expansion as there were more than one copy of *cas2* gene in the clade I MAGs (Table S3) and phage was predicted in 66.67% of the MAGs of clade I (Table S3). In addition, multicopy genes could be predicted in MAGs of clade I (Table S3). Although studies indicated that a larger genome of microbes endows greater versatility (Nielsen et al., 2021), genome size as a trait was nearly independent from cell size and growth rate of various bacterial ecological types (Nielsen et al., 2021; Westoby et al., 2021). Considering the high diversity of isolation sources, the roles of KSB1 bacteria might be differentiated notably.

### Relative abundance of KSB1 in different niches

**Figure 2:**
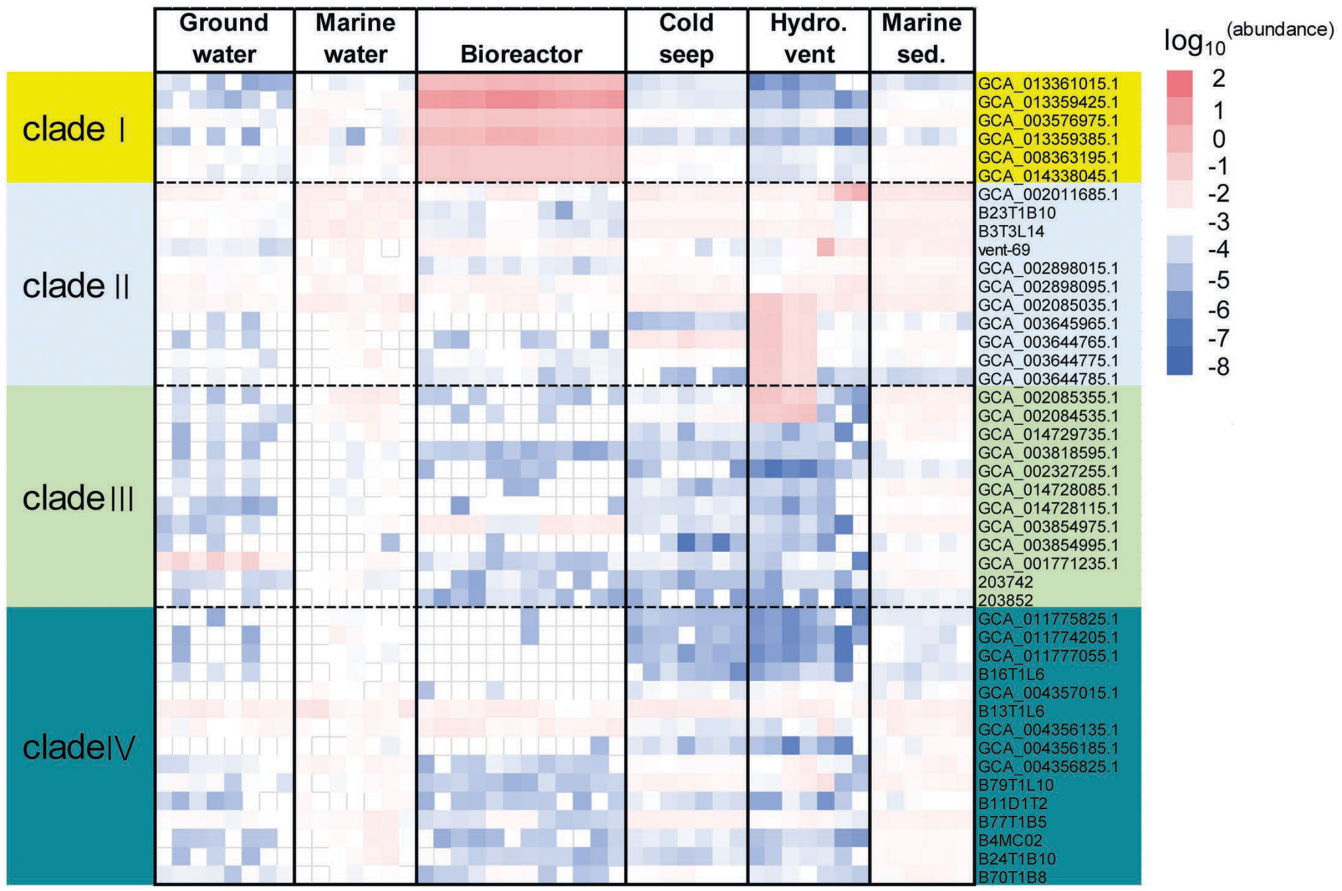
Relative abundance of KSB1 genomes from different clades in metagenomes. The coverage of each MAG by metagenomic reads as a proxy of relative abundance was calculated by CoverM and then transformed using log_10_. Marine sed., marine sediment; Hydro. vent, hydrothermal vent.

To evaluate the distribution of KSB1 in different environments, relative abundance of KSB1 was assessed using percentage of 16S miTags and coverage of KSB1 MAGs in metagenomes. The metagenome raw data for the calculation were downloaded from NCBI (Table S4). Our results showed that the relative abundance of MAGs belonging to clade I was most abundant in bioreactor (Fig. 2; Table S5), indicating that KSB1 of clade I may be likely one of the representative bacterial groups in wastewater treatment. Clade IV was more prevalent in marine sediment than other environments. All the four clades were present in sea water and were more abundant than in groundwater (Fig. 2).

To examine vertical distribution of KSB1 in marine waters, relative abundance of KSB1 16S miTags in those of Tara Ocean project (Moran, 2015) was calculated. Clade II was relatively more abundant in the oceans, compared to the other clades (Fig. S2). Clade II was the most abundant at 5-m surface layer (0.65%) than other zones (Fig. S2; Table S5), whereas clade IV was detectable in marine waters below 200 m (Fig. S2; Table S5). Nevertheless, the low abundance of KSB1 in the Tara Ocean data suggests limited distribution of the KSB1 bacteria in oxic marine waters.

### Carbohydrate-active enzymes (CAZYmes) in KSB1 genomes

**Figure 3:**
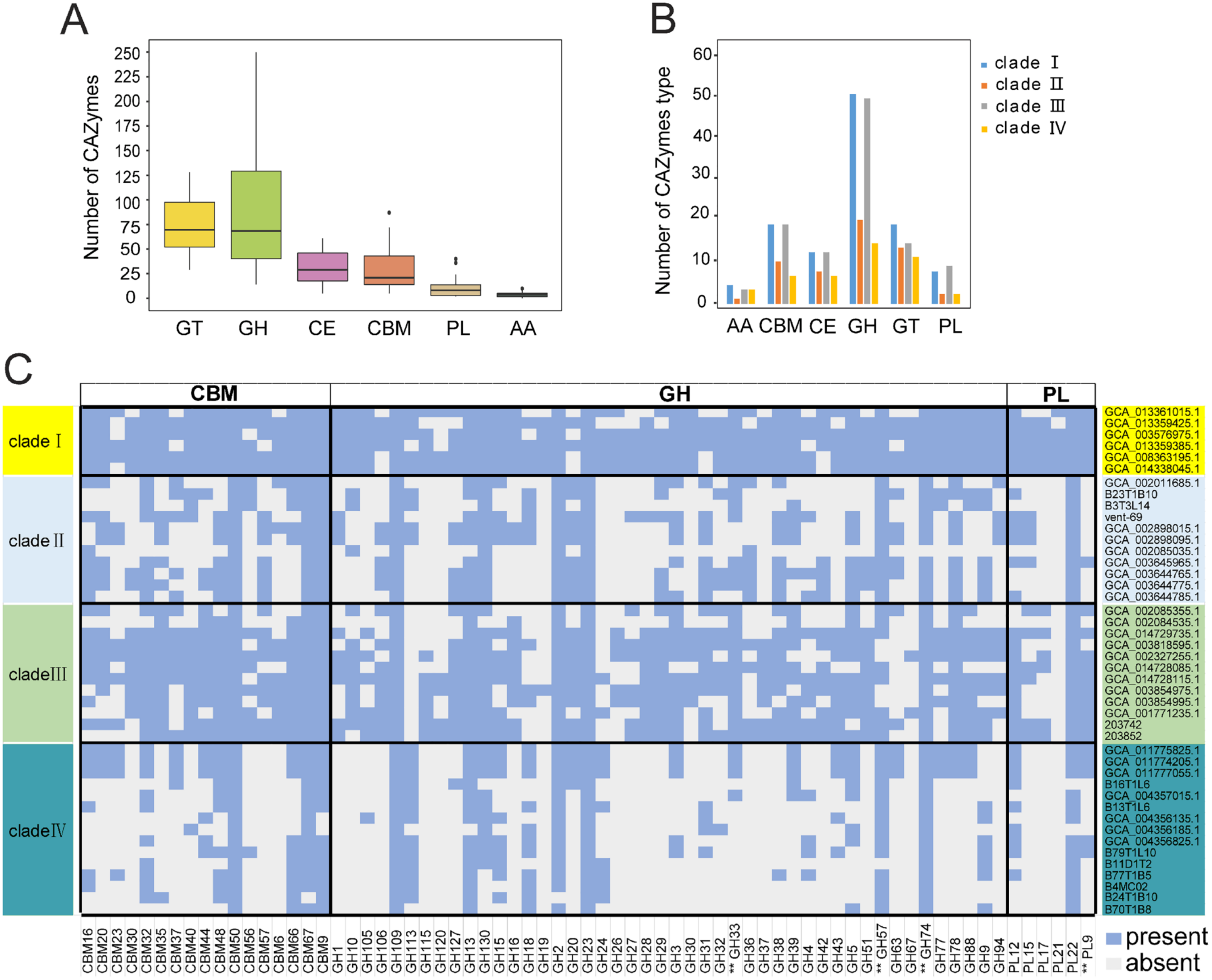
Carbohydrate-active enzymes (CAZymes) encoded by KSB1 genomes. (A) Number of CAZyme classes in KSB1 MAGs. (B) Number of CAZyme subfamilies in predicted proteins of KSB1 clades. (C) Heatmap illustrating presence (light blue) or absence (light grey) of CAZymes in each MAG. GH, glycoside hydrolases; PL, polysaccharide lyases; GT, glycosyl transferases; CE, carbohydrate esterases; CBM, carbohydrate-binding modules; AA, auxiliary activities. CAZymes marked with double asterisks refer to CAZyme associated with a potential secretion signal.

KSB1 may be broadly distributed due to their potential functions on degrading complex carbohydrates. An analysis of CAZymes in the 44 KSB1 MAGs revealed diverse GH and GT classes (Fig. 3A). Particularly, GH109, GH23, GT4, GT2, CE10 (esterase) and CBM50 (LysM) could be identified in all MAGs (Table S6). GH23 includes lytic transglycosylases with helice D, F and beta-sheet, acting on peptidoglycan to cleave the glycosidic linkage between N-acetylglucosaminyl and N-actetylmuramoyl residues to produce cyclic 1,6-anhydro-N-acetylmuramic acid (anhMurNAc) (Harding et al., 2020; Scheurwater, Reid & Clarke, 2008). CBM50 can interact with chitin and peptidoglycan (Bertucci et al., 2019). GT2 and GT4, including α-glucosyltransferase and chitin synthase, were dominant families of GTs (Bohra, Dafale & Purohit, 2019). These results indicate that the five types of CAZyme subfamilies were found across KSB1 and potentially allow them to obtain nutrients from various organic substrates such as chitin, peptidoglycan and other components of cell wall.

Clades I and III contained more CAZYmes of different subfamilies, compared to the other clades (Fig. 3B; Table S6), which indicated that clades I and III of KSB1 encode a broader repertoire of CAZymes than clades II and IV apart from CAZYmes involved in auxiliary activities (AA). Furthermore, CBM, GH and PL were absent in many MAGs of clades II and IV (Fig. 3C; Table S6). Notably, CAZymes with a potential secretion signal (Dombrowski, Teske & Baker, 2018) were different in distribution among four clades. GH28, GH33 and PL9 were absent in clades II and IV (Fig. 3C) and this seems to be a result of low availability of labile carbohydrates in deep-sea hydrothermal vent or hadal sediments (Richardson et al., 1995). As a contrast, such CAZYmes were more common in KSB1 MAGs of clade I that were identified in bioreactor sludge enriched with abundant and complex organic carbon sources (Mata et al., 2020) (Fig. 3C). The high variety of CAZymes in clade III agrees with their diverse isolation sources as shown in Fig. 1D.

### Core metabolic genes detected in KSB1 genomes

**Figure 4:**
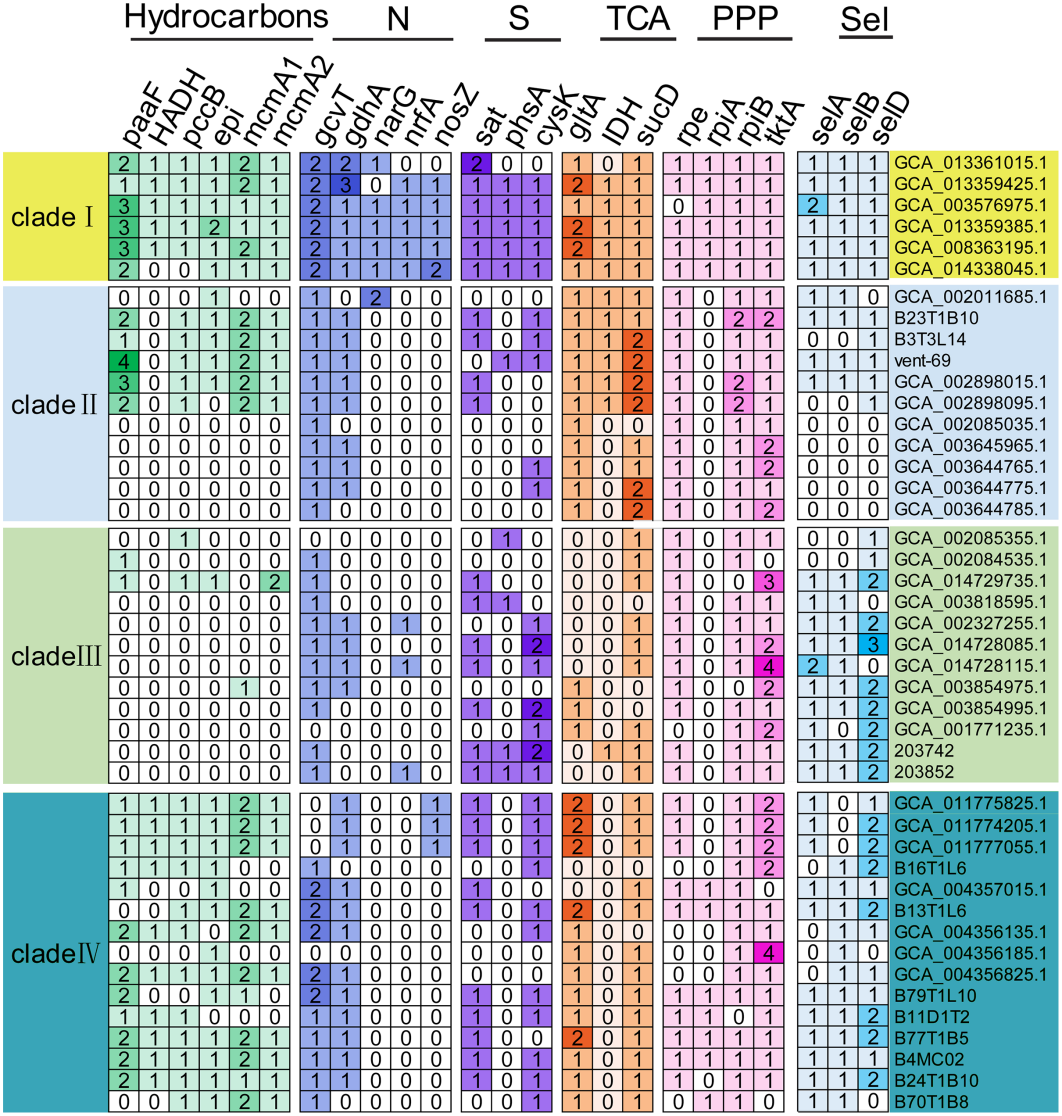
Genes involved in core metabolism predicted in KSB1 genomes. Copy number of functional genes related to carbon, nitrogen, sulfur, and selenate metabolism pathway was displayed in the heatmap for the KSB1 clades. TCA, tricarboxylic acid cycle; PPP, pentose phosphate pathway; Sel, selenate metabolism.

The functional genes responsible for utilization of carbohydrates, associated to nitrogen, sulfur and selenate metabolisms, were detected in the KSB1 MAGs (Fig. 4). The core genes of *gltA* and *sucD* related to citric acid cycle (TCA) were present in four clades of KSB1 (Fig. 4). The core genes of *rpe*, *rpiA*, *rpiB* and *tktA* involved in pentose phosphate pathway (PPP) were also present in four clades of KSB1 (Fig. 4). These results indicate that central carbon metabolism is nearly complete in KSB1. About half of MAGs of clade II encoded genes involved in hydrocarbon metabolism (Fig. 4), although the MAGs mainly came from hydrothermal environment (Fig. 1D). There has been reported abiogenic hydrocarbon in hydrothermal system (McDermott et al., 2015; Proskurowski et al., 2008), which may be the carbon source for growth of KSB1 that encoded genes associated to hydrocarbon metabolism. KSB1 MAGs harbored more than one copy of *paaF*, *pccB*, *epi*, *mcmA1* and *mcmA2* genes that are involved in short-chain hydrocarbon transformation (Fig. 4). They were abundantly present in clades I and IV of KSB1. It had been reported that an operon consisting of 14 *paa* genes encode enzymes to degrade phenylacetate (Teufel et al., 2010). PaaABCDE catalyze phenylacetyl-CoA to ring 1,2-epoxyphenylacetyl-CoA (Teufel et al., 2010). Paa converts 3-hydroxyadipyl-CoA to 3-oxoadipyl-CoA with NADH as a byproduct (Teufel et al., 2010). PaaABH coding genes were detected in clade I of KSB1 (Table S7), which indicated that the clade I might catabolize phenylacetic acid or act on the intermediates of the whole pathway to obtain energy. However, most of the *paa* genes (*paaZCDEGIJKXY*) were not identified in clade I; catalytic experiment of Paa complex of clade I in phenylacetic acid utilization is needed in future work. *mcmA1* and *mcmA2* encode α-subunit of methylmalonyl-CoA mutase that takes part in propanoate pathway (Han et al., 2013). PccB participates in the conversion of propionyl-CoA to methylmalonyl-CoA (Wongkittichote, Ah Mew & Chapman, 2017), which might be subsequently converted by McmA1 or McmA2 to enter TCA cycle (Bobik & Rasche, 2001). EPI (MCEE) is a methylmalonyl-CoA epimerase responsible for degradation of odd chain-length fatty acids and branched-chain amino acids (Dobson et al., 2006). The interconversion of D- and L-methylmalonyl-CoA might be performed by EPI, which is a key step in propanoyl-CoA to succinyl-CoA for TCA cycle (Dobson et al., 2006). Since all these genes have been identified in the KSB1 genomes, the metabolism of propionyl-CoA to succinyl-CoA might be employed by KSB1 for short hydrocarbons degradation into TCA cycle (Fig. 4), which was similar to previous report that KSB1 was involved in anaerobic degradation of hydrocarbon in hydrothermal sediments (Dombrowski et al., 2017). HADH that was present in clades I and IV is a 3-hydroxyacyl-CoA dehydrogenase gene involved in fatty acids metabolism as described in a previous study that KSB1 has the capacity for beta-oxidation (Baker et al., 2015). Coupled with the other core genes such as *pccB*, *mcmA1*, *mcmA2* and *epi*, the result suggests that the clades I and IV might break down fatty acids and hydrocarbons for energy.

*narG*, *nrfA* and *nosZ* for denitrification were only present in clade I of KSB1 (Fig. 4). Nitrate could be reduced to nitrite by nitrite oxidoreductase encoded by *narG* and the following reduction of nitrite to ammonia could be finished with the function of *nrfA* (Giblin et al., 2013). This indicates that MAGs affiliated with KSB1 clade I may be involved in dissimilatory nitrate reduction in bioreactor, which was not reported previously (Youssef et al., 2019). *nosZ* gene involved in the last step of denitrification to produce nitrogen gas (Giblin et al., 2013), was revealed in five out of six MAGs of clade I (Fig. 4). However, the other genes related to denitrification (*nirK* or *norBC*) could not be found in MAGs of clade I (Table S7). The genes only identified in the clade I might be the results of lateral gene transfer as indicated by the genome expansion (Fig. 1B). Phylogenetic trees were built to examine the origin of NarG and NrfA predicted in KSB1 clade I. Our results showed that the NarG sequences of KSB1 clade I were grouped with the homologs from *Acidobacteria*, Rokubacteria and NC10, while the NrfA sequences were adjacent to those derived from *Anaerolineae*, ‘*Candidatus* Jettenia’ and ‘*Candidatus* Brocadia’ (Fig. S3). In addition, the NosZ sequences of KSB1 clade I were approximate to the homologs from *Ignavibacteria* and were associated with sec-type signal peptide (Fig. S4; Table S8) (Jones et al., 2013). There were some genes responsible for sulfur metabolism such as *sat* (sulfate adenylyltransferase), *phsA* (polysulfide reductase chain A) and *cysK* (cysteine synthase) in the KSB1 MAGs (Fig. 4). *sat* and *cysK* were identified in almost all KSB1 except clade II. Particularly, *sat*, encoding a protein responsible for activation of inorganic sulfate, was used for catalysis of sulfate to adenylyl sulfate (Fauque & Barton, 2012). PhsA, mostly present in clade I, converts thiosulfate to sulfide for synthesis of cysteine by CysK (Chen et al., 2018b). This suggests that KSB1 might take part in the assimilatory sulfate reduction in bioreactor. *selA*, *selB* and *selD* genes were predicted in MAGs of KSB1 except clade II (Fig. 4). SelA (selenocysteine synthase) and SelD (selenophosphate synthase) were required for selenocysteine synthesis (Leinfelder et al., 1990). SelB is a Sec-specific elongation factor for the incorporation of selenocysteine into proteins (Sheppard et al., 2008). With the presence of the three genes in KSB1, the biosynthesis of selenocysteine may take place in KSB1. Selenocysteine can be incorporated into proteins as well when *selC* (selenocysteyl-tRNAsec) is mutant (Zorn et al., 2013). The protein containing selenocysteine (selenoproteins) functions in antioxidant system and redox regulation of signal pathway (Zhang et al., 2020). The prevalence of *selABD* genes and absence of *selC* gene (Table S7) in KSB1 genomes suggest that selenoproteins might be biosynthesized by KSB1 to resist stress in diverse niches.

### Metabolism reconstruction of KSB1

**Figure 5:**
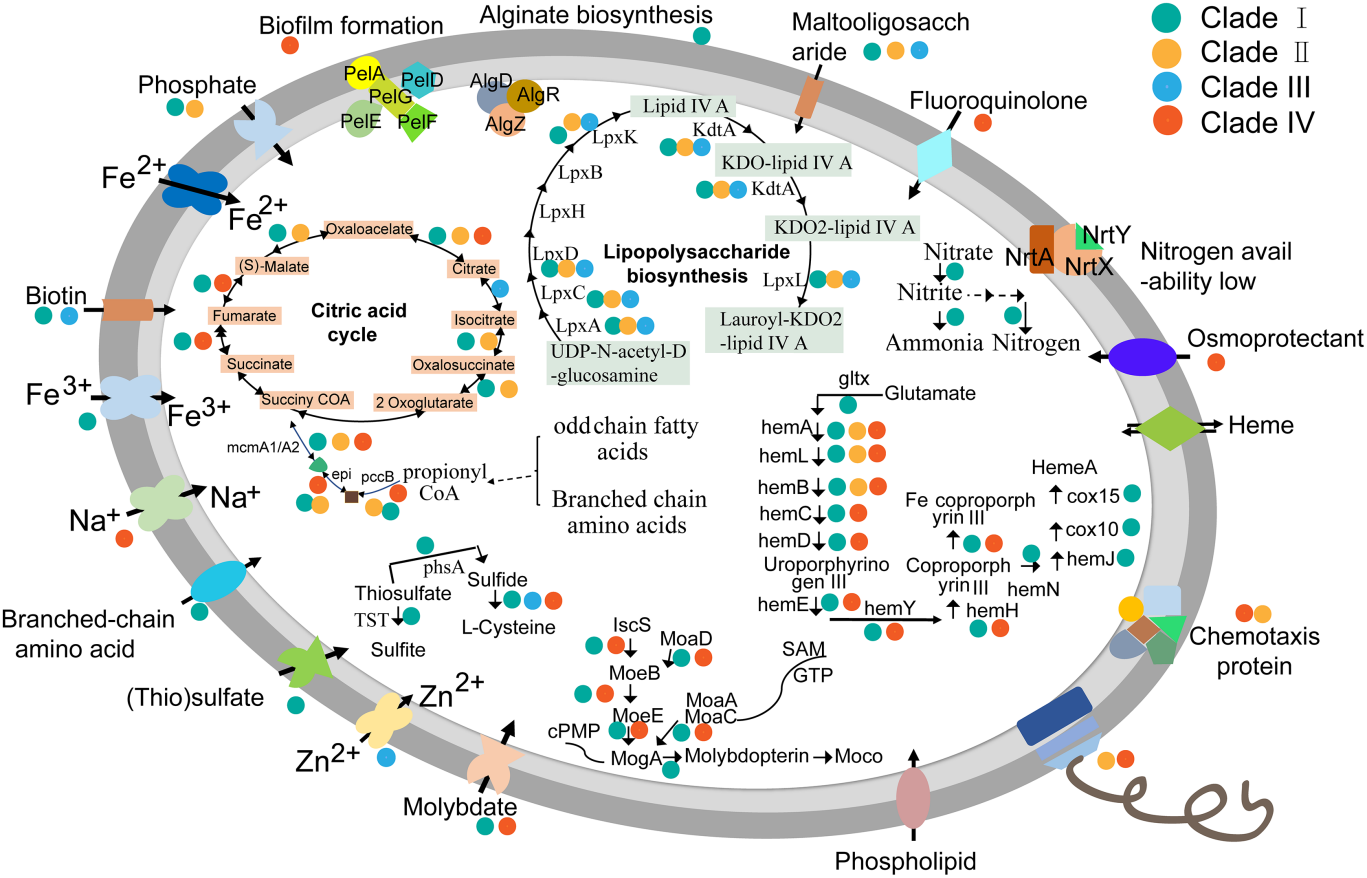
Schematic metabolism mode of KSB1. Metabolism pathways were reconstructed based on KEGG annotation. The dots with different colors refer to KSB1 clade IDs. The genes that could be detected in all clades were not associated with any dot.

To learn more about characteristics of KSB1 lifestyle, metabolism reconstruction was performed. Almost all genes related to flagellar biosynthetic proteins, flagellar assembly proteins and other flagellar structure proteins were identified in KSB1 genomes affiliated with clades II and IV (Fig. 5; Table S7). In addition, the stator element of the flagellar motor complex MotA, methyl-accepting chemotaxis protein MCP and the proteins of two-component system controlling chemotaxis (Table S7) were encoded by clades II and IV. The flagellar and chemotaxis responsible for motility might be useful for KSB1 clades II and IV to explore nutrients in oligotrophic deep-sea environment. The KSB1 genomes of clade I contain the genes coding for some ABC transporters for uptake of biotin, ferrous and ferric ion, branched-chain amino acids, molybdate and maltooligosaccharide (Fig. 5; Table S7). These transporters may be employed by KSB1 clade I to import organic and inorganic nutrients from bioreactor used for waste treatment. The (thio)sulfate transporter related gene was only present in clade I MAGs (Fig. 5). Thiosulfate can be oxidized to sulfite by thiosulfate/3-mercaptopyruvate sulfurtransferase (TST) or reduced to sulfide by thiosulfate reductase/polysulfide reductase chain A (PHSA) in anoxic conditions (Jorgensen, 1990). This indicates that KSB1 of clade I might take part in “thiosulfate shunt” in the bioreactor sludge. These pathways associated to sulfur might occur in the KSB1 inhabiting in sulfur-rich black mud (Tanner et al., 2000), anoxic zone with a low-H_2_S rather than high-H_2_S concentration (Ley et al., 2006). The genes named *cydA* and *cydB* were also predicted in clade I (Table S7). *cydAB* encode subunits of cytochrome *bd* that is expressed under low oxygen condition (Borisov et al., 2011; Kranz et al., 1983), which indicates that KSB1 could live in O_2_-limited environments (Borisov et al., 2011). Nitrogen regulation genes (*ntrY* and *ntrX*) were all detected in KSB1 genomes of clade I (Table S7), which is likely required for controlling the level of cytoplasmic nitrogen (Carrica et al., 2012). In addition, the key genes (*algD*, *algR* and *algZ*) of alginate biosynthesis (Leech et al., 2008; Wu et al., 2015) were detected in KSB1 genomes of clade I (Fig. 5; Table S7), suggesting that they might be involved in alginate biosynthesis as one strategy for keeping carbon storage.

The molybdate transporter genes such as *modA* (coding for molybdate transport system substrate-binding protein) and *modB* (coding for molybdate transport system permease protein) were identified in KSB1 genomes of clades I and IV (Fig. 5; Table S7). In addition, the genes involved in Moco (molybdenum cofactor) biosynthesis (Nichols & Rajagopalan, 2005) were also identified (Fig. 5; Table S7). The function of Moco in KSB1 genomes is not clear yet, although it has been reported that the impairment of Moco biosynthesis affected mobility, anaerobic respiration and biofilm formation (Andreae, Titball & Butler, 2014). Moco required for molybdoenzymes was probably important for bacteria to adapt in harsh or dramatically changing redox condition (Leimkuhler & Iobbi-Nivol, 2016), and therefore these genes may encode proteins for survival of KSB1 in bioreactor or deep-sea sediments.

When the lipopolysaccharide (LPS) biosynthesis pathway was examined, the genes (*lpxABCDKL* and *kdtA*) involved in this pathway were identified in almost all KSB1 genomes except clade IV (Fig. 5; Table S7). The encoded proteins might use UDP-N-acetyl-alpha-D-glucosamine as substrate for biosynthesis of lauroyl-KDO2-lipid IV(A) and LPS (Wang, Quinn & Yan, 2015). The potential capacity of LPS biosynthesis suggests that KSB1 might be gram-negative bacteria. The pellicle (PEL) polysaccharide-dependent biofilm formation related genes (*pelADEFG*) (Whitfield et al., 2020a; Whitfield et al., 2020b) were present in KSB1 genomes of clade IV (Fig. 5; Table S7). Biofilm is a special colony that contains microbial cells and extracellular matrix (Davies et al., 1998). The presence of these genes in the clade IV KSB1 genomes suggests that biofilm may be important for their survival in nutrient-poor deep-sea sediments.

A set of genes involved in heme biosynthesis and ferrous iron transportation were identified in KSB1 genomes of clades I and IV (Fig. 5; Table S7). The Fe-coproporphyrin III biosynthesis might be finished in KSB1 genomes of clades I and IV due to the presence of *hemABCDELHY* (Fig. 5; Table S7). The presence of *hemN* in clade I indicated that they may biosynthesize heme A, a component of cytochrome oxidases (Hederstedt, 2012). It had been reported that *hemA* and *hemL* were necessary for heme biosynthesis and electron transfer in anaerobic respiratory metabolism (Frankenberg, Moser & Jahn, 2003; Zumft, 1997). Collectively, this might be a strategy of KSB1 clade I inhabiting bioreactor sludge to obtain energy under anaerobic conditions by denitrification using heme nitrite reductase with iron regulating heme biosynthesis.

## Conclusions

This study has examined the phylogenetic relationships, distribution, genomic features and potential metabolism pathways of the candidate bacterial phylum KSB1. KSB1 was divided into four phylogenetic clades featured with different gene profiles and niche adaptation. The clades were significantly different when compared with each other, which indicated that the versatile metabolism of KSB1 inhabiting different niches. Clade I of KSB1 might be one of critical players in wastewater treatment bioreactors with O_2_-limited or anoxic conditions as suggested by its high incidence in sludge and broad functional potentials (*e.g*., diverse carbon degradation, nitrate reduction, assimilatory sulfate reduction and alginate biosynthesis). However, the high metabolic diverseity of clade I was not observed in clade II inhabiting in hydrothermal vents and relying on energy that might be obtained by abiogenic hydrocarbon metabolism. The clade III may encode many classes of CAZymes, which may allow them to synthesize or break down complex carbohydrates and sugars in diverse niches rather than fatty acid or short-chain hydrocarbons in special environment as occurring in other clades. Clade IV may have the capability of molybdate transportation and molybdenum cofactor biosynthesis as clade I for adaptation in extreme conditions. Overall, the KSB1 bacteria are probably heterotrophs depending on hydrocarbons as all known autotrophic carbon fixation pathways could not be identified in their genomes. Nevertheless, more data and experiments are expected to support the functional potentials of KSB1 predicted by this study.

## Supplemental Information

10.7717/peerj.13241/supp-1 Supplemental Information 1 All KSB1 genomes collected from different public databases and MAGs binned in this study.

10.7717/peerj.13241/supp-2 Supplemental Information 2 GTDB taxonomy and quality score of KSB1.

10.7717/peerj.13241/supp-3 Supplemental Information 3 The multicopy genes, transposase and phage predicted of KSB1 clade I.

10.7717/peerj.13241/supp-4 Supplemental Information 4 Accession number of metagenomes from different niches.

10.7717/peerj.13241/supp-5 Supplemental Information 5 Relative abundance of KSB1 in different niches assessed by 16S miTAG of Tara Ocean and raw reads of metagenome download from public database.

10.7717/peerj.13241/supp-6 Supplemental Information 6 Presence count in each MAG and percentage of each clade of CAZYmes of KSB1.

10.7717/peerj.13241/supp-7 Supplemental Information 7 KEGG annotation of each MAG of KSB1.

10.7717/peerj.13241/supp-8 Supplemental Information 8 Signal peptide predicted online of nosZ with gram-positive or gram-negative model by signalP5.0 online.

10.7717/peerj.13241/supp-9 Supplemental Information 9 Phylogenomics tree reflecting KSB1 position in FCB superphylum. The phylogenetic tree was built by using concatenated aligned conserved proteins of KSB1 and reference genomes. High quality MAGs of Therrabacteria and Proteobacteria were used as the outgroup genomes.

10.7717/peerj.13241/supp-10 Supplemental Information 10 Distribution of KSB1 in marine water. Relative abundance of KSB1 was calculated as a percentage of KSB1 16S miTags in metagenomes of the Tara Ocean project. The depth range of the metagenomes is between 5 and 1,000 m.

10.7717/peerj.13241/supp-11 Supplemental Information 11 The phylogenetic tree of proteins encoded by horizontal transferred *narG* (A) and *nrfA* (B) genes. The NarG (A) and NrfA (B) phylogenetic trees were built by IQ-TREE with MFP+LM model. The black dots with different size scales on the branches represent the bootstrap values obtained with 1,000 replicates. The protein sequences of NarG and NrfA identified in MAGs of KSB1 clade I were marked in light blue.

10.7717/peerj.13241/supp-12 Supplemental Information 12 The phylogenetic tree and signal peptide of NosZ proteins. (A) The rooted phylogenetic tree of NosZ proteins was built by IQ-TREE with MFP+LM model. The black dots with different size scales on branches represent the bootstrap values obtained with 1,000 replicates. The protein sequences of NosZ identified in MAGs of KSB1 clade I were marked in orange; (B) The leaves in different colors in the unrooted NosZ phylogenetic tree represent the types of signal peptide predicted by signalP 5.0 online with gram positive or negative model.

10.7717/peerj.13241/supp-13 Supplemental Information 13 The alignment files of trees in this study and the 16S sequences of KSB1.

## Additional Information and Declarations

### Data Availability

The following information was supplied regarding data availability: The total eleven MAGs of KSB1 binned from the Mariana sediments and the Mid-Atlantic Ridge hydrothermal sediment were submitted to the National Omics Data Encyclopedia (NODE) with OEP002159 as project accession number and OER184014 as run ID.

## References

[ref-1] Al-Shayeb B, Sachdeva R, Chen LX, Ward F, Munk P, Devoto A, Castelle CJ, Olm MR, Bouma-Gregson K, Amano Y, He C, Meheust R, Brooks B, Thomas A, Lavy A, Matheus-Carnevali P, Sun C, Goltsman DSA, Borton MA, Sharrar A, Jaffe AL, Nelson TC, Kantor R, Keren R, Lane KR, Farag IF, Lei S, Finstad K, Amundson R, Anantharaman K, Zhou J, Probst AJ, Power ME, Tringe SG, Li WJ, Wrighton K, Harrison S, Morowitz M, Relman DA, Doudna JA, Lehours AC, Warren L, Cate JHD, Santini JM, Banfield JF (2020). Clades of huge phages from across Earth’s ecosystems. Nature.

[ref-2] Ali M, Shaw DR, Albertsen M, Saikaly PE (2020). Comparative genome-centric analysis of freshwater and marine anammox cultures suggests functional redundancy in nitrogen removal processes. Frontiers in Microbiology.

[ref-3] Almeida A, Mitchell AL, Boland M, Forster SC, Gloor GB, Tarkowska A, Lawley TD, Finn RD (2019). A new genomic blueprint of the human gut microbiota. Nature.

[ref-4] Anantharaman K, Brown CT, Hug LA, Sharon I, Castelle CJ, Probst AJ, Thomas BC, Singh A, Wilkins MJ, Karaoz U, Brodie EL, Williams KH, Hubbard SS, Banfield JF (2016). Thousands of microbial genomes shed light on interconnected biogeochemical processes in an aquifer system. Nature Communications.

[ref-5] Andreae CA, Titball RW, Butler CS (2014). Influence of the molybdenum cofactor biosynthesis on anaerobic respiration, biofilm formation and motility in *Burkholderia thailandensis*. Research in Microbiology.

[ref-6] Aramaki T, Blanc-Mathieu R, Endo H, Ohkubo K, Kanehisa M, Goto S, Ogata H (2020). KofamKOALA: KEGG Ortholog assignment based on profile HMM and adaptive score threshold. Bioinformatics.

[ref-7] Baker BJ, Lazar CS, Teske AP, Dick GJ (2015). Genomic resolution of linkages in carbon, nitrogen, and sulfur cycling among widespread estuary sediment bacteria. Microbiome.

[ref-8] Bankevich A, Nurk S, Antipov D, Gurevich AA, Dvorkin M, Kulikov AS, Lesin VM, Nikolenko SI, Pham S, Prjibelski AD, Pyshkin AV, Sirotkin AV, Vyahhi N, Tesler G, Alekseyev MA, Pevzner PA (2012). SPAdes: a new genome assembly algorithm and its applications to single-cell sequencing. Journal of Computational Biology.

[ref-9] Bertucci M, Calusinska M, Goux X, Rouland-Lefevre C, Untereiner B, Ferrer P, Gerin PA, Delfosse P (2019). Carbohydrate hydrolytic potential and redundancy of an anaerobic digestion microbiome exposed to acidosis, as uncovered by metagenomics. Applied and Environmental Microbiology.

[ref-10] Bobik TA, Rasche ME (2001). Identification of the human methylmalonyl-CoA racemase gene based on the analysis of prokaryotic gene arrangements. Implications for decoding the human genome. Journal of Biological Chemistry.

[ref-11] Bohra V, Dafale NA, Purohit HJ (2019). Understanding the alteration in rumen microbiome and CAZymes profile with diet and host through comparative metagenomic approach. Archives of Microbiology.

[ref-12] Borisov VB, Gennis RB, Hemp J, Verkhovsky MI (2011). The cytochrome bd respiratory oxygen reductases. Biochimica Et Biophysica Acta.

[ref-13] Capella-Gutierrez S, Silla-Martinez JM, Gabaldon T (2009). trimAl: a tool for automated alignment trimming in large-scale phylogenetic analyses. Bioinformatics.

[ref-14] Cardinali-Rezende J, Pereira ZL, Sanz JL, Chartone-Souza E, Nascimento AM (2012). Bacterial and archaeal phylogenetic diversity associated with swine sludge from an anaerobic treatment lagoon. World Journal of Microbiology and Biotechnology.

[ref-15] Carrica MD, Fernandez I, Marti MA, Paris G, Goldbaum FA (2012). The NtrY/X two-component system of Brucella spp. acts as a redox sensor and regulates the expression of nitrogen respiration enzymes. Molecular Microbiology.

[ref-16] Chaumeil PA, Mussig AJ, Hugenholtz P, Parks DH (2019). GTDB-Tk: a toolkit to classify genomes with the Genome Taxonomy Database. Bioinformatics.

[ref-17] Chen Z, Zhang X, Li H, Liu H, Xia Y, Xun L (2018b). The complete pathway for thiosulfate utilization in *Saccharomyces cerevisiae*. Applied and Environmental Microbiology.

[ref-18] Chen S, Zhou Y, Chen Y, Gu J (2018a). Fastp: an ultra-fast all-in-one FASTQ preprocessor. Bioinformatics.

[ref-19] Cui G, Zhou Y, Li W, Gao Z, Huang J, Wang Y (2021). A novel bacterial phylum that participates in carbon and osmolyte cycling in the Challenger Deep sediments. Environmental Microbiology.

[ref-20] Dalcin Martins P, Frank J, Mitchell H, Markillie LM, Wilkins MJ (2019). Wetland sediments host diverse microbial taxa capable of cycling alcohols. Applied and Environmental Microbiology.

[ref-21] Davies DG, Parsek MR, Pearson JP, Iglewski BH, Costerton JW, Greenberg EP (1998). The involvement of cell-to-cell signals in the development of a bacterial biofilm. Science.

[ref-22] Dobson CM, Gradinger A, Longo N, Wu X, Leclerc D, Lerner-Ellis J, Lemieux M, Belair C, Watkins D, Rosenblatt DS, Gravel RA (2006). Homozygous nonsense mutation in the MCEE gene and siRNA suppression of methylmalonyl-CoA epimerase expression: a novel cause of mild methylmalonic aciduria. Molecular Genetics and Metabolism.

[ref-23] Dombrowski N, Seitz KW, Teske AP, Baker BJ (2017). Genomic insights into potential interdependencies in microbial hydrocarbon and nutrient cycling in hydrothermal sediments. Microbiome.

[ref-24] Dombrowski N, Teske AP, Baker BJ (2018). Expansive microbial metabolic versatility and biodiversity in dynamic Guaymas Basin hydrothermal sediments. Nature Communications.

[ref-25] Eddy SR (1995). Multiple alignment using hidden Markov models. Third International Conference on Intelligent Systems for Molecular Biology.

[ref-26] Fauque GD, Barton LL (2012). Hemoproteins in dissimilatory sulfate- and sulfur-reducing prokaryotes. Recent Advances in Microbial Oxygen-binding Proteins.

[ref-27] Fish JA, Chai BL, Wang Q, Sun YN, Brown CT, Tiedje JM, Cole JR (2013). FunGene: the functional gene pipeline and repository. Frontiers in Microbiology.

[ref-28] Frankenberg N, Moser J, Jahn D (2003). Bacterial heme biosynthesis and its biotechnological application. Applied Microbiology and Biotechnology.

[ref-29] Giblin AE, Tobias CR, Song B, Weston N, Banta GT, Rivera-Monroy VH (2013). The importance of dissimilatory nitrate reduction to ammonium (DNRA) in the nitrogen cycle of coastal ecosystems. Oceanography.

[ref-30] Gish W, States DJ (1993). Identification of protein coding regions by database similarity search. Nature Genetics.

[ref-31] Han J, Hou J, Zhang F, Ai G, Li M, Cai S, Liu H, Wang L, Wang Z, Zhang S, Cai L, Zhao D, Zhou J, Xiang H (2013). Multiple propionyl coenzyme A-supplying pathways for production of the bioplastic poly(3-hydroxybutyrate-co-3-hydroxyvalerate) in Haloferax mediterranei. Applied and Environmental Microbiology.

[ref-32] Harding CJ, Huwiler SG, Somers H, Lambert C, Ray LJ, Till R, Taylor G, Moynihan PJ, Sockett RE, Lovering AL (2020). A lysozyme with altered substrate specificity facilitates prey cell exit by the periplasmic predator *Bdellovibrio bacteriovorus*. Nature Communications.

[ref-33] Hederstedt L (2012). Heme A biosynthesis. Biochimica Et Biophysica Acta.

[ref-34] Huang JM, Wang Y (2020). Genomic differences within the phylum Marinimicrobia: from waters to sediments in the Mariana Trench. Marine Genomics.

[ref-35] Hyatt D, Chen GL, Locascio PF, Land ML, Larimer FW, Hauser LJ (2010). Prodigal: prokaryotic gene recognition and translation initiation site identification. BMC Bioinformatics.

[ref-36] Jones CM, Graf DRH, Bru D, Philippot L, Hallin S (2013). The unaccounted yet abundant nitrous oxide-reducing microbial community: a potential nitrous oxide sink. ISME Journal.

[ref-37] Jorgensen BB (1990). A thiosulfate shunt in the sulfur cycle of marine sediments. Science.

[ref-38] Katoh K, Standley DM (2013). MAFFT multiple sequence alignment software version 7: improvements in performance and usability. Molecular Biology and Evolution.

[ref-39] Kranz RG, Barassi CA, Miller MJ, Green GN, Gennis RB (1983). Immunological characterization of an *Escherichia coli* strain which is lacking cytochrome-D. Journal of Bacteriology.

[ref-40] Leech AJ, Sprinkle A, Wood L, Wozniak DJ, Ohman DE (2008). The NtrC family regulator AlgB, which controls alginate biosynthesis in mucoid *Pseudomonas aeruginosa*, binds directly to the algD promoter. Journal of Bacteriology.

[ref-41] Leimkuhler S, Iobbi-Nivol C (2016). Bacterial molybdoenzymes: old enzymes for new purposes. FEMS Microbiology Reviews.

[ref-42] Leinfelder WFK, Veprek B, Zehelein E, Böck A (1990). In vitro synthesis of selenocysteinyl-tRNAUCA from seryl-tRNAUCA: involvement and characterization of the *selD* gene product. Proceedings of the National Academy of Sciences of the United States of America.

[ref-43] Ley RE, Harris JK, Wilcox J, Spear JR, Miller SR, Bebout BM, Maresca JA, Bryant DA, Sogin ML, Pace NR (2006). Unexpected diversity and complexity of the Guerrero Negro hypersaline microbial mat. Applied and Environmental Microbiology.

[ref-44] Li H, Durbin R (2009). Fast and accurate short read alignment with Burrows-Wheeler transform. Bioinformatics.

[ref-45] Li W, Godzik A (2006). Cd-hit: a fast program for clustering and comparing large sets of protein or nucleotide sequences. Bioinformatics.

[ref-46] Li H, Handsaker B, Wysoker A, Fennell T, Ruan J, Homer N, Marth G, Abecasis G, Durbin R, Genome Project Data Processing Subgroup (2009). The sequence alignment/map format and SAMtools. Bioinformatics.

[ref-47] Mata SN, Santos TD, Cardoso LG, Andrade BB, Duarte JH, Costa JAV, de Souza CO, Druzian JI (2020). Spirulina sp. LEB 18 cultivation in a raceway-type bioreactor using wastewater from desalination process: production of carbohydrate-rich biomass. Bioresource Technology.

[ref-48] McDermott JM, Seewald JS, German CR, Sylva SP (2015). Pathways for abiotic organic synthesis at submarine hydrothermal fields. Proceedings of the National Academy of Sciences of the United States of America.

[ref-49] Minh BQ, Schmidt HA, Chernomor O, Schrempf D, Woodhams MD, von Haeseler A, Lanfear R (2020). IQ-TREE 2: new models and efficient methods for phylogenetic inference in Genomic Era. Molecular Biology and Evolution.

[ref-50] Moran MA (2015). The global ocean microbiome. Science.

[ref-51] Newton ILG, Bordenstein SR (2011). Correlations between bacterial ecology and mobile DNA. Current Microbiology.

[ref-52] Nichols JD, Rajagopalan KV (2005). In vitro molybdenum ligation to molybdopterin using purified components. Journal of Biological Chemistry.

[ref-53] Nielsen DA, Fierer N, Geoghegan JL, Gillings MR, Gumerov V, Madin JS, Moore L, Paulsen IT, Reddy TBK, Tetu SG, Westoby M (2021). Aerobic bacteria and archaea tend to have larger and more versatile genomes. Oikos.

[ref-54] Parks DH, Imelfort M, Skennerton CT, Hugenholtz P, Tyson GW (2015). CheckM: assessing the quality of microbial genomes recovered from isolates, single cells, and metagenomes. Genome Research.

[ref-55] Proskurowski G, Lilley MD, Seewald JS, Fruh-Green GL, Olson EJ, Lupton JE, Sylva SP, Kelley DS (2008). Abiogenic hydrocarbon production at Lost City hydrothermal field. Science.

[ref-56] Richardson MD, Briggs KB, Bowles FA, Tietjen JH (1995). A depauperate benthic assemblage from the nutrient-poor sediments of the Puerto-Rico Trench. Deep Sea Research Part I: Oceanographic Research Papers.

[ref-57] Sanderson H, Ortega-Polo R, Zaheer R, Goji N, Amoako KK, Brown RS, Majury A, Liss SN, McAllister TA (2020). Comparative genomics of multidrug-resistant Enterococcus spp. isolated from wastewater treatment plants. BMC Microbiology.

[ref-58] Scheurwater E, Reid CW, Clarke AJ (2008). Lytic transglycosylases: bacterial space-making autolysins. International Journal of Biochemistry & Cell Biology.

[ref-59] Sheppard K, Yuan J, Hohn MJ, Jester B, Devine KM, Soll D (2008). From one amino acid to another: tRNA-dependent amino acid biosynthesis. Nucleic Acids Research.

[ref-60] Tanner MA, Everett CL, Coleman WJ, Yang MM, Youvan D (2000). Complex microbial communities inhabiting sulfide-rich black mud from marine coastal environments. Biotechnol Alia.

[ref-61] Tatusov RL, Galperin MY, Natale DA, Koonin EV (2000). The COG database: a tool for genome-scale analysis of protein functions and evolution. Nucleic Acids Research.

[ref-62] Teufel R, Mascaraque V, Ismail W, Voss M, Perera J, Eisenreich W, Haehnel W, Fuchs G (2010). Bacterial phenylalanine and phenylacetate catabolic pathway revealed. Proceedings of the National Academy of Sciences of the United States of America.

[ref-63] Uritskiy GV, DiRuggiero J, Taylor J (2018). MetaWRAP—a flexible pipeline for genome-resolved metagenomic data analysis. Microbiome.

[ref-64] Wang X, Quinn PJ, Yan A (2015). Kdo2-lipid A: structural diversity and impact on immunopharmacology. Biological Reviews.

[ref-65] Westoby M, Nielsen DA, Gillings MR, Litchman E, Madin JS, Paulsen IT, Tetu SG (2021). Cell size, genome size, and maximum growth rate are near-independent dimensions of ecological variation across bacteria and archaea. Ecology and Evolution.

[ref-66] Whitfield GB, Marmont LS, Bundalovic-Torma C, Razvi E, Roach EJ, Khursigara CM, Parkinson J, Howell PL (2020a). Discovery and characterization of a Gram-positive Pel polysaccharide biosynthetic gene cluster. PLOS Pathogens.

[ref-67] Whitfield GB, Marmont LS, Ostaszewski A, Rich JD, Whitney JC, Parsek MR, Harrison JJ, Howell PL (2020b). Pel polysaccharide biosynthesis requires an inner membrane complex comprised of PelD, PelE, PelF, and PelG. Journal of Bacteriology.

[ref-68] Wongkittichote P, Ah Mew N, Chapman KA (2017). Propionyl-CoA carboxylase—a review. Molecular Genetics and Metabolism.

[ref-69] Wu DQ, Cheng H, Duan Q, Huang W (2015). Sodium houttuyfonate inhibits biofilm formation and alginate biosynthesis-associated gene expression in a clinical strain of Pseudomonas aeruginosa in vitro. Experimental and Therapeutic Medicine.

[ref-70] Wu H, Zhang Z, Hu SN, Yu J (2012). On the molecular mechanism of GC content variation among eubacterial genomes. Biology Direct.

[ref-71] Xu H, Luo X, Qian J, Pang X, Song J, Qian G, Chen J, Chen S (2012). FastUniq: a fast de novo duplicates removal tool for paired short reads. PLOS ONE.

[ref-72] Yasir M (2018). Analysis of bacterial communities and characterization of antimicrobial strains from cave microbiota. Brazilian Journal of Microbiology.

[ref-73] Youssef NH, Farag IF, Hahn CR, Jarett J, Becraft E, Eloe-Fadrosh E, Lightfoot J, Bourgeois A, Cole T, Ferrante S, Truelock M, Marsh W, Jamaleddine M, Ricketts S, Simpson R, McFadden A, Hoff W, Ravin NV, Sievert S, Stepanauskas R, Woyke T, Elshahed M (2019). Genomic characterization of candidate division LCP-89 reveals an atypical cell wall structure, microcompartment production, and dual respiratory and fermentative capacities. Applied and Environmental Microbiology.

[ref-74] Zhang Y, Roh YJ, Han SJ, Park I, Lee HM, Ok YS, Lee BC, Lee SR (2020). Role of selenoproteins in redox regulation of signaling and the antioxidant system: a review. Antioxidants.

[ref-75] Zorn M, Ihling CH, Golbik R, Sawers RG, Sinz A (2013). Selective selc-independent selenocysteine incorporation into formate dehydrogenases. PLOS ONE.

[ref-76] Zumft WG (1997). Cell biology and molecular basis of denitrification. Microbiology and Molecular Biology Reviews.

