# Supplementary material for: Phylogeny, distribution and potential metabolism of candidate bacterial phylum KSB1
Source: PeerJ. 2022 Apr 12;10:e13241. doi: 10.7717/peerj.13241 (PMC9012183; doi:10.7717/peerj.13241)
Supplement: Supplemental Information 9 — The phylogenetic tree was built by using concatenated aligned conserved proteins of KSB1 and reference genomes. High quality MAGs of Therrabacteria and Proteobacteria were used as the outgroup genomes. [file peerj-10-13241-s009.pdf]

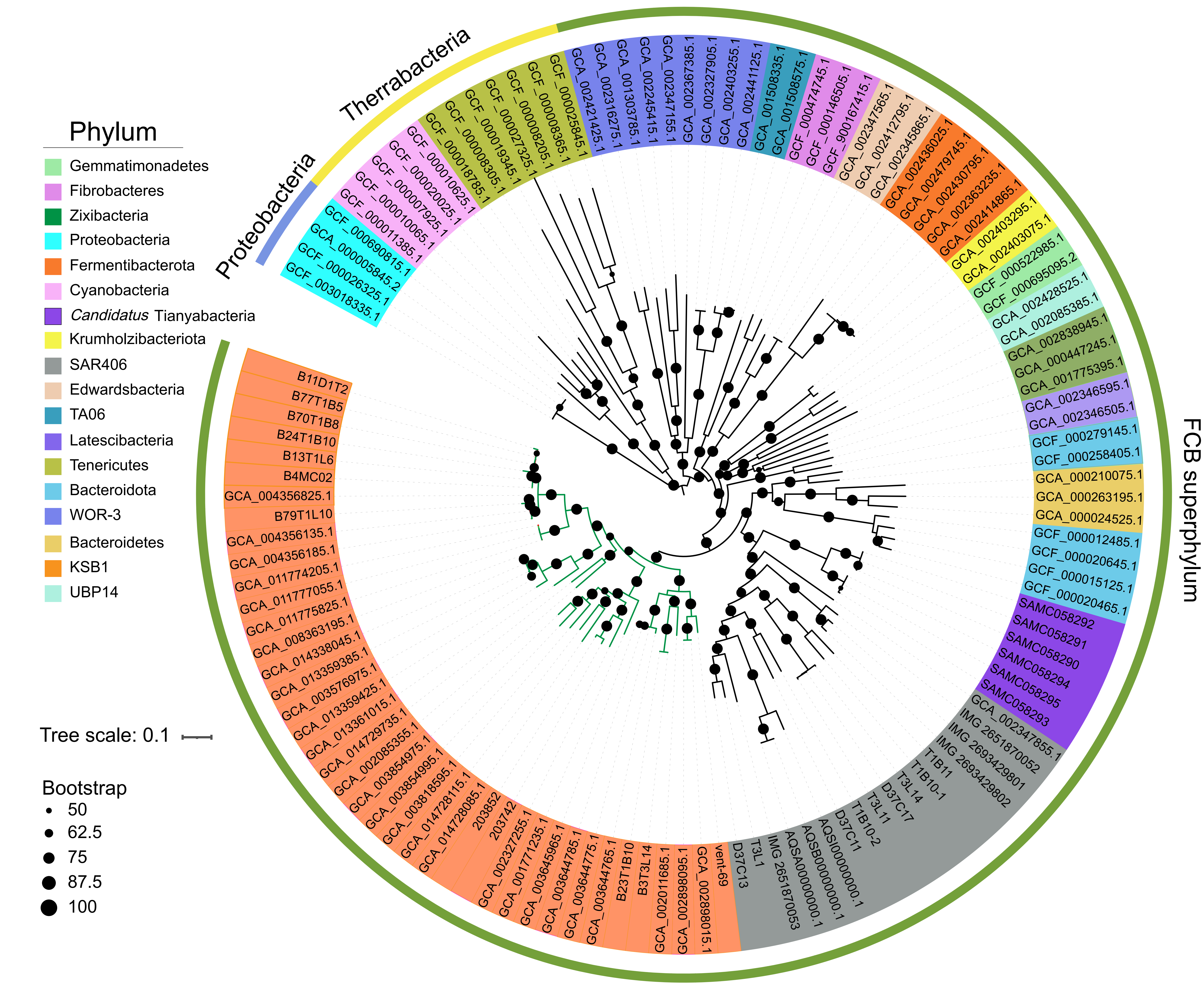
Phylum
Gemmatimonadetes
Fibrobacteres
Zixibacteria
Proteobacteria
Fermentibacterota
Cyanobacteria
Candidatus Tianyabacteria
Krumholzibacteriota
SAR406
Edwardsbacteria
TA06
Latescibacteria
Tenericutes
Bacteroidota
WOR-3
Bacteroidetes
KSB1
UBP14
Tree scale: 0.1
Bootstrap
50
62.5
75
87.5
100
Proteobacteria
Therrabacteria
FCB superphylum
GCF_003018335.1
GCF_000026325.1
GCA_000005845.2
GCF_000690815.1
GCF_000011385.1
GCF_000010065.1
GCF_000007925.1
GCF_000020025.1
GCF_000010625.1
GCF_000018785.1
GCF_000008305.1
GCF_000019345.1
GCF_000027325.1
GCF_000008205.1
GCF_000008365.1
GCF_000025845.1
GCA_002421425.1
GCA_002316275.1
GCA_001303785.1
GCA_002245415.1
GCA_002347155.1
GCA_002367385.1
GCA_002327905.1
GCA_002403255.1
GCA_002441125.1
GCA_001508335.1
GCA_001508575.1
GCF_000474745.1
GCF_000146505.1
GCF_900167415.1
GCA_002347565.1
GCA_002412795.1
GCA_002345865.1
GCA_002436025.1
GCA_002479745.1
GCA_002430795.1
GCA_002363235.1
GCA_002414865.1
GCA_002403295.1
GCA_002403075.1
GCF_000522985.1
GCF_000695095.2
GCA_002428525.1
GCA_002085385.1
GCA_002838945.1
GCA_000447245.1
GCA_001775395.1
GCA_002346595.1
GCA_002346505.1
GCF_000279145.1
GCF_000258405.1
GCA_000210075.1
GCA_000263195.1
GCA_000024525.1
GCF_000012485.1
GCF_000020645.1
GCF_000015125.1
GCF_000020465.1
SAMC058292
SAMC058291
SAMC058290
SAMC058294
SAMC058295
SAMC058293
GCA_002347855.1
IMG 2651870052
IMG 2693429801
IMG 2693429802
T1B11
T1B10-1
T3L14
D37C17
T3L11
T1B10-2
D37C11
AQSI00000000.1
AQSB00000000.1
AQSA00000000.1
IMG 2651870053
T3L1
D37C13
vent-69
GCA_002898015.1
GCA_002898095.1
GCA_002011685.1
B3T3L14
B23T1B10
GCA_003644765.1
GCA_003644775.1
GCA_003644785.1
GCA_003645965.1
GCA_001771235.1
GCA_002327255.1
203742
203852
GCA_014728085.1
GCA_014728115.1
GCA_003818595.1
GCA_003854995.1
GCA_003854975.1
GCA_002085355.1
GCA_014729735.1
GCA_013361015.1
GCA_013359425.1
GCA_003576975.1
GCA_013359385.1
GCA_014338045.1
GCA_008363195.1
GCA_011775825.1
GCA_011777055.1
GCA_011774205.1
GCA_004356185.1
GCA_004356135.1
B79T1L10
GCA_004356825.1
B4MC02
B13T1L6
B24T1B10
B70T1B8
B77T1B5
B11D1T2
