# Supplementary figures and images for: Phylogeny, distribution and potential metabolism of candidate bacterial phylum KSB1
Source: PeerJ. 2022 Apr 12;10:e13241. doi: 10.7717/peerj.13241 (PMC9012183; doi:10.7717/peerj.13241)

relative abundance (%)

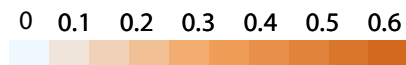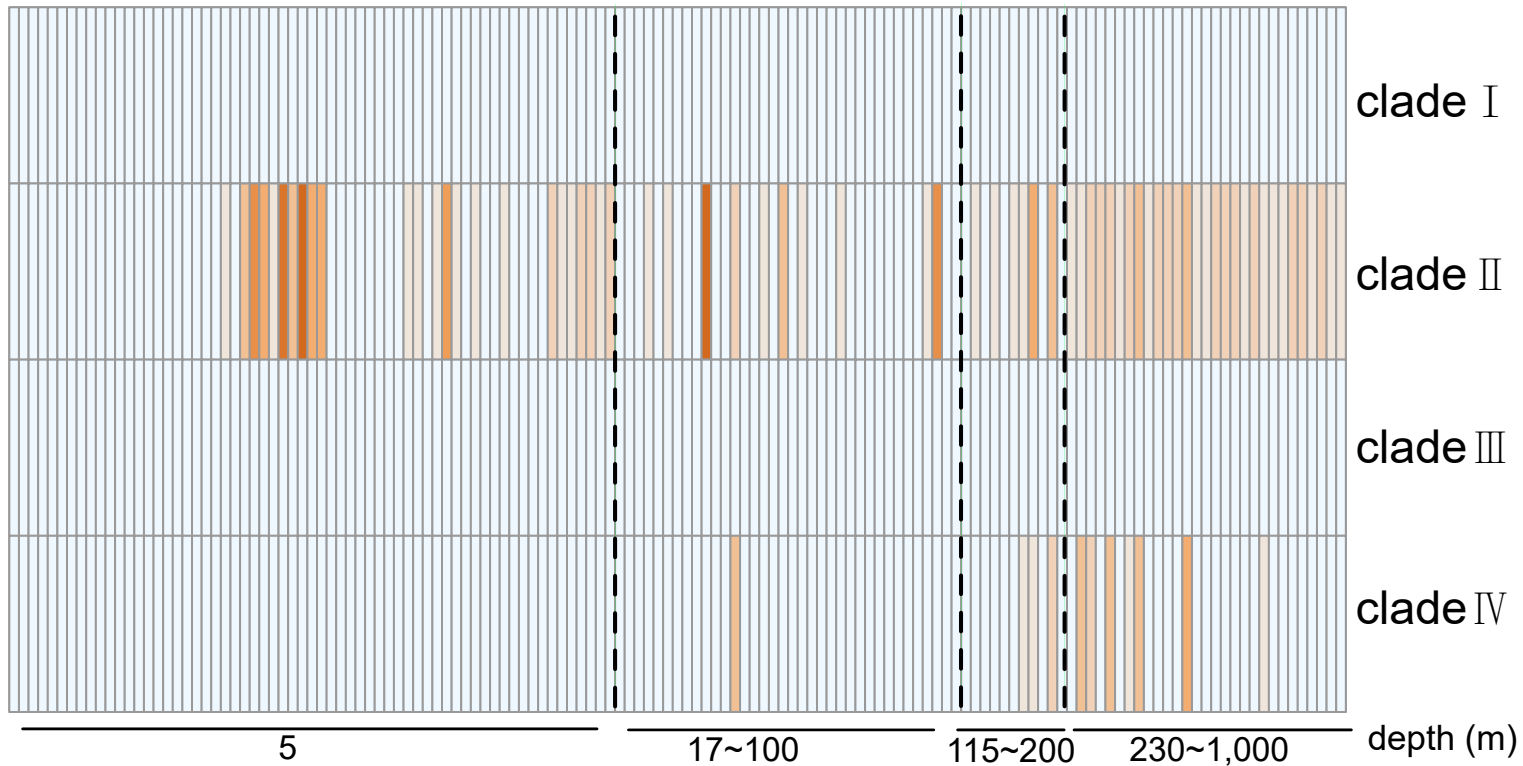

Supplement: Supplemental Information 10 — Relative abundance of KSB1 was calculated as a percentage of KSB1 16S miTags in metagenomes of the Tara Ocean project. The depth range of the metagenomes is between 5 and 1,000 m. [file peerj-10-13241-s010.pdf]

A

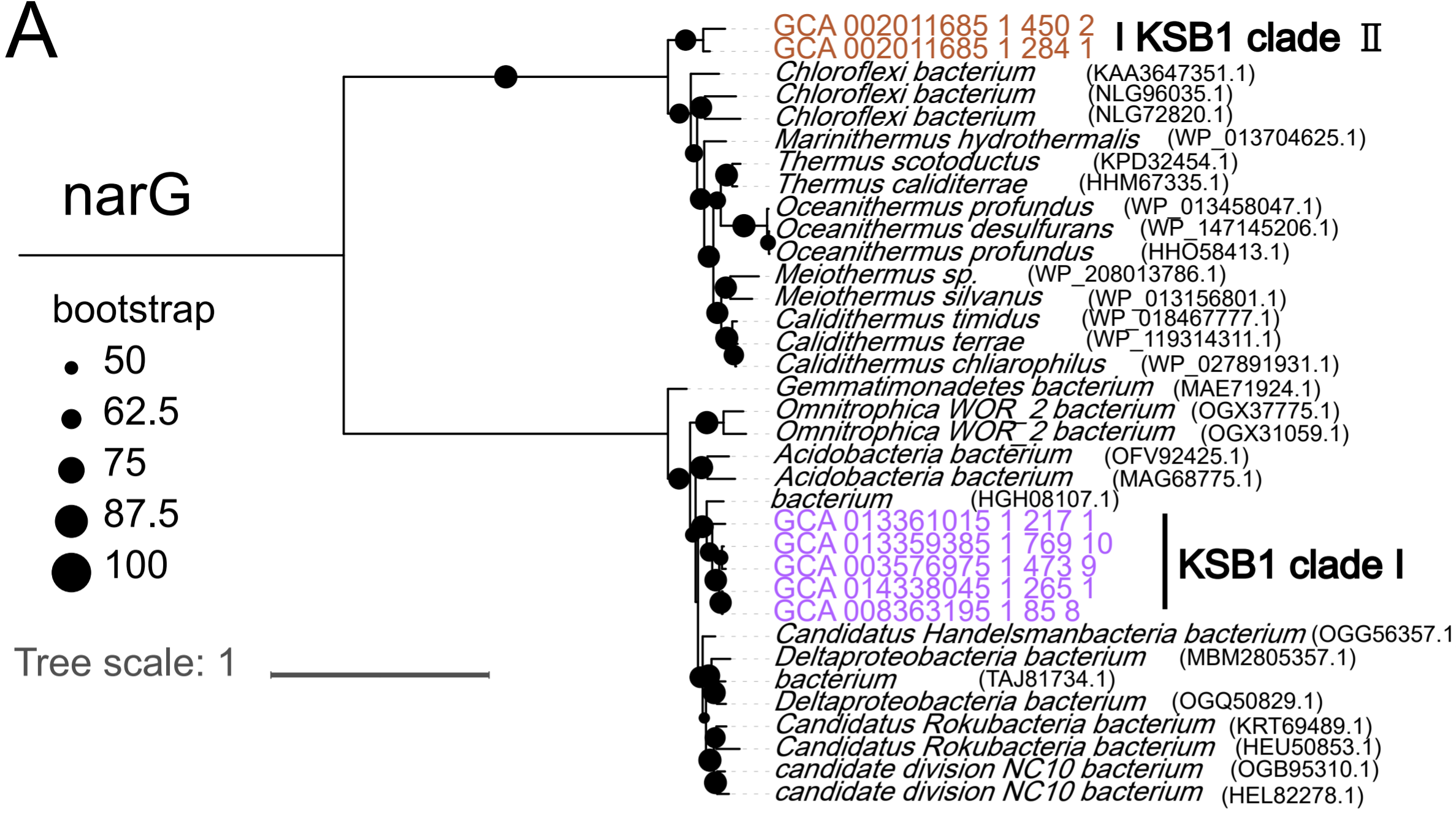

B

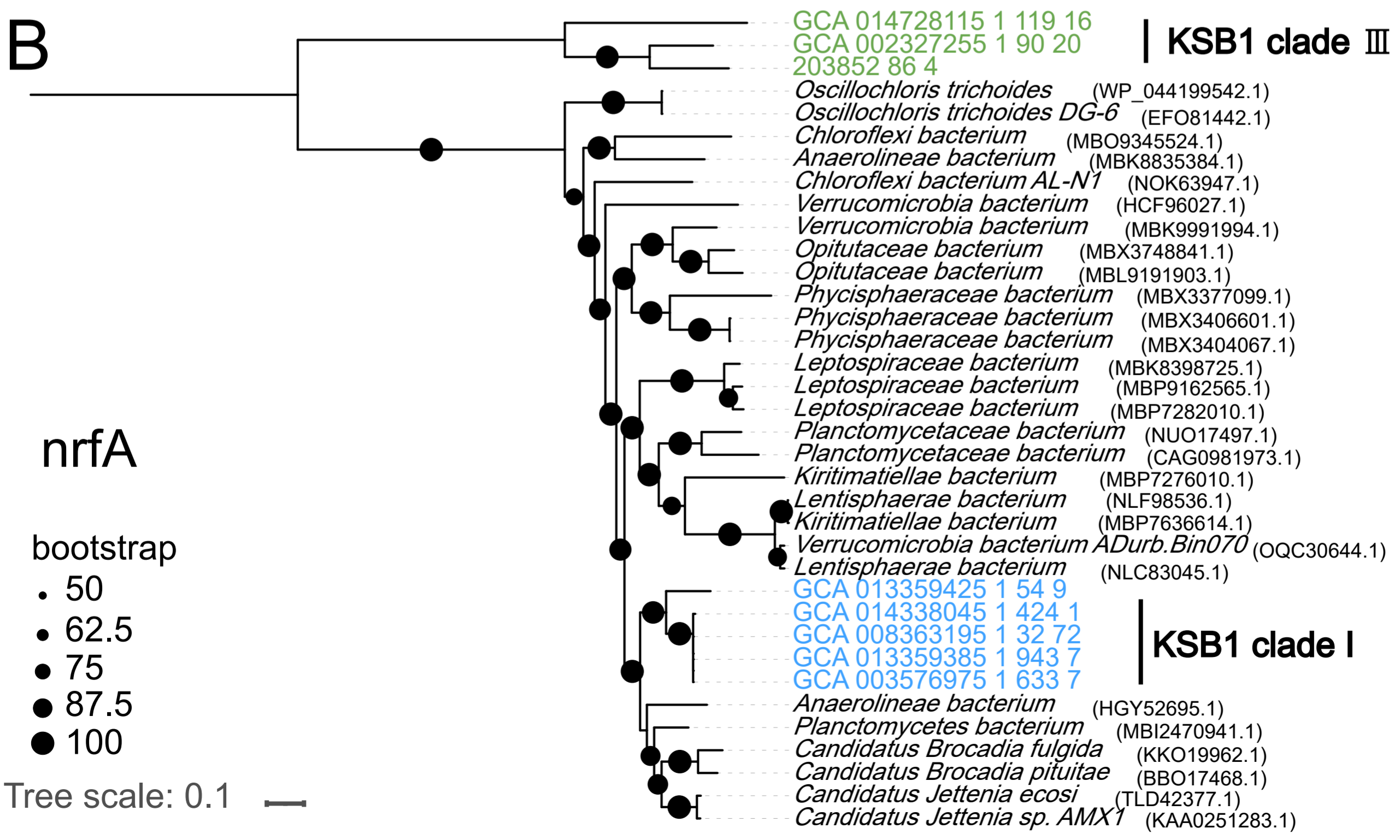

Supplement: Supplemental Information 11 — The NarG (A) and NrfA (B) phylogenetic trees were built by IQ-TREE with MFP+LM model. The black dots with different size scales on the branches represent the bootstrap values obtained with 1,000 replicates. The protein sequences of NarG and NrfA identified in MAGs of KSB1 clade I were marked in light blue. [file peerj-10-13241-s011.pdf]

A

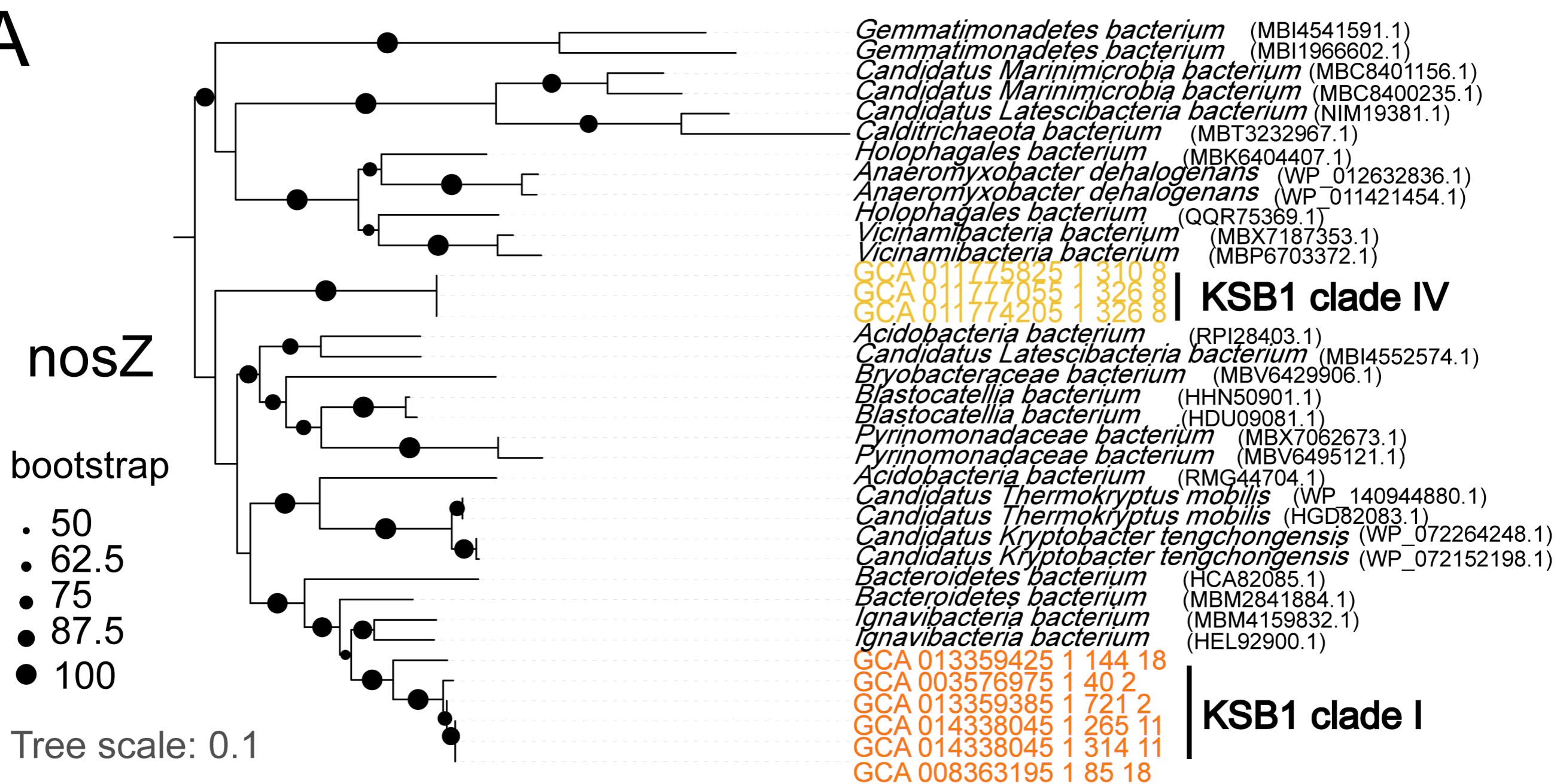

B

Tree scale: 1

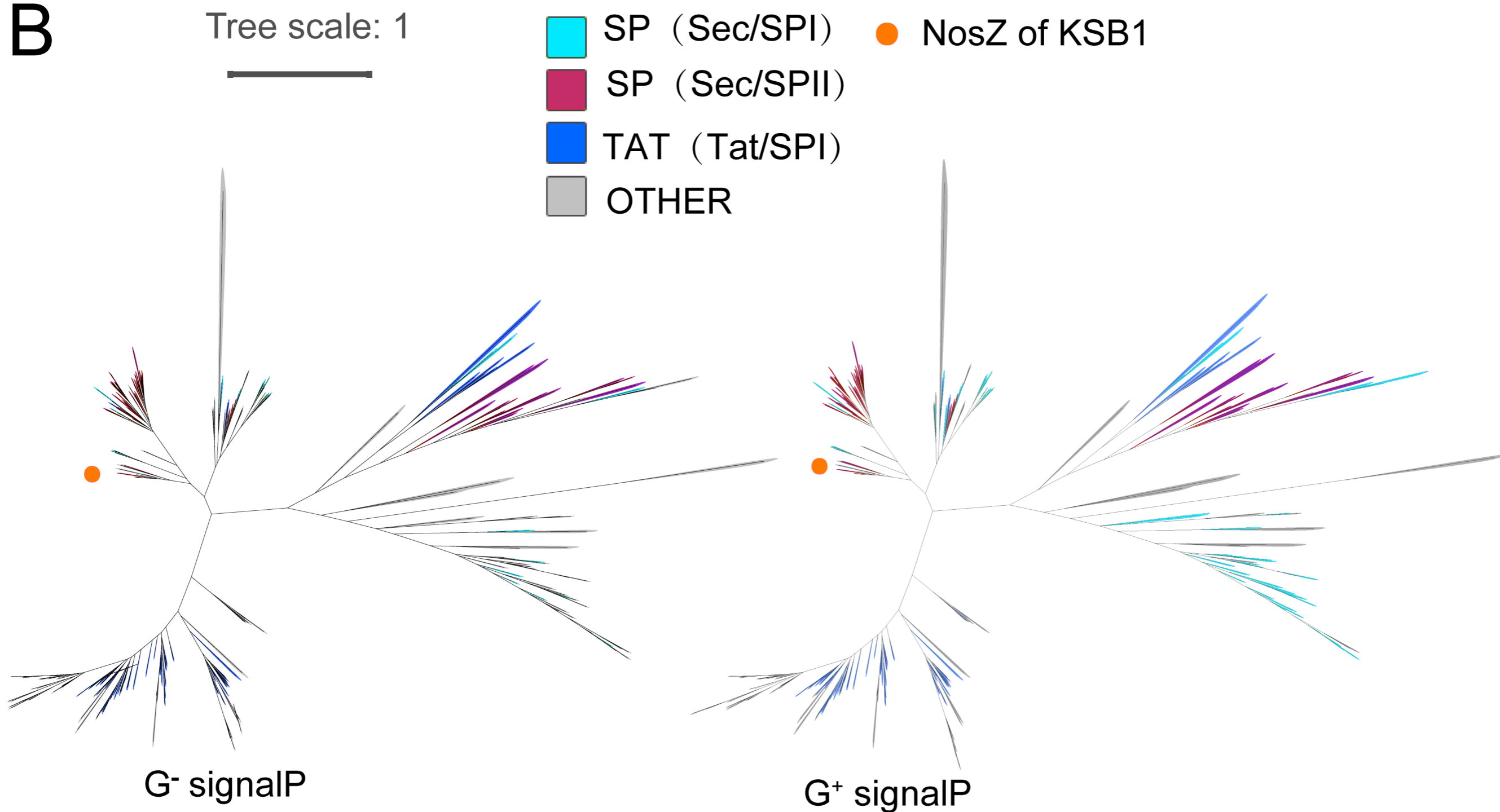

Supplement: Supplemental Information 12 — (A) The rooted phylogenetic tree of NosZ proteins was built by IQ-TREE with MFP+LM model. The black dots with different size scales on branches represent the bootstrap values obtained with 1,000 replicates. The protein sequences of NosZ identified in MAGs of KSB1 clade I were marked in orange; (B) The leaves in different colors in the unrooted NosZ phylogenetic tree represent the types of signal peptide predicted by signalP 5.0 online with gram positive or negative model. [file peerj-10-13241-s012.pdf]
